# A multiwavelength emission detector for analytical ultracentrifugation†

**DOI:** 10.1039/c9na00487d

**Published:** 2019-10-04

**Authors:** Simon E. Wawra, Georgy Onishchukov, Maria Maranska, Siegfried Eigler, Johannes Walter, Wolfgang Peukert

**Affiliations:** Institute of Particle Technology (LFG), Friedrich-Alexander-Universität Erlangen-Nürnberg (FAU) Cauerstrasse 4 91058 Erlangen Germany wolfgang.peukert@fau.de; Institute for Chemistry and Biochemistry, Freie Universität Berlin Takustraße 3 14195 Berlin Germany; Interdisciplinary Center for Functional Particle Systems (FPS), Friedrich-Alexander-Universität Erlangen-Nürnberg (FAU) Haberstrasse 9a 91058 Erlangen Germany

## Abstract

In this study, a new detector for multiwavelength emission analytical ultracentrifugation (MWE-AUC) is presented, which allows measuring size- or composition-dependent fluorescence properties of nanoparticle ensembles. Validation of the new setup is carried out *via* comparison to a benchtop photoluminescence spectrometer and the established extinction-based multiwavelength analytical ultracentrifuge (MWL-AUC). The results on fluorescent proteins and silica particles demonstrate that the new device not only correctly reproduces sedimentation and diffusion coefficients of the particles but provides also meaningful fluorescence spectra. As an application example for a sample exhibiting a broad particle size distribution, spectra and size of graphene oxide nanoplatelets are extracted simultaneously. Narrowly distributed CdSe/ZnS quantum dots showing size- and structure-dependent shifts of their fluorescence spectra are analyzed as well. The combination of MWE- and MWL-AUC provides a comprehensive framework for the optical characterization for nanoparticles and macromolecules in terms of their extinction and emission properties.

## Abstract 2

The new analytical ultracentrifugation setup allows coupling hydrodynamic, thermodynamic and emission properties of nanoparticles and macromolecules.

## Graphical abstract

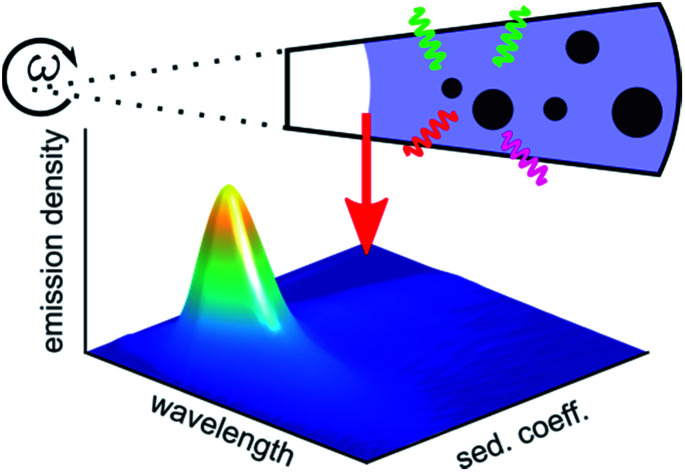

## Introduction

Particle design is of ever increasing importance. In order to conduct fundamental studies or improve product properties, extensive characterization methods are needed. This is especially important for size- and shape-dependent optical properties of nanoparticles, like fluorescence of silicon nanocrystals^1^ or extinction of quantum dots.^2^ Most characterization methods, lacking the possibility of fractionation, struggle with polydisperse samples and are unable to combine information on multiple sample properties. To overcome these limitations,^3–5^ analytical ultracentrifugation (AUC) is used more and more for comprehensive characterization of particles.^3,5,6^ From the temporal and radial evolution of the particle concentration during sedimentation in a centrifugal field, hydro- and thermodynamic properties can be retrieved. Throughout the long history of AUC, several different detection concepts have been developed to access the sedimentation dynamics. The first detection principle was based on light absorption,^7^ then the detection of the concentration-dependent refraction index *via* an interferometer was put into place^8^ as well as the detection of its radial gradient *via* Schlieren optics.^9^ Additionally, integral fluorescence intensities of particle and protein ensembles were used for analysis in AUC.^10–12^ Combining spectral information at several wavelengths^13^ led to the development of multiwavelength extinction AUC (MWL-AUC)^14^ extending AUC analysis towards complex and often coupled size-dependent hydrodynamic, thermodynamic and optical particle properties opening up vast new possibilities, including *e.g.* the identification of different species^4,5^ or the determination of two-dimensional size distributions of anisotropic plasmonic particles.^3^ Even wide property distributions can now be analyzed by gravitational sweep experiments at constant radial position.^4,15,16^ So far unexplored further potential in terms of accessible data lies in size-dependent fluorescence light detection in dependence of the excitation wavelength. At the moment, any spectral information in the formerly commercially sold fluorescence AUC cannot be acquired due to the usage of a photomultiplier with a longpass or broadband bandpass spectral filter. Without the spectral information at hand, species labelled with different fluorescent dyes cannot be easily discriminated. Therefore, multi-component detection of protein complexes can currently only be achieved by using photo-switchable proteins.^17^ Nevertheless, the method is very popular as it allows to study protein interactions at nanomolar concentrations.^18^ Apart from the patented concept^19^ itself, multiwavelength emission analytical ultracentrifuge (MWE-AUC) was not demonstrated yet. In this contribution, we present a novel setup for multiwavelength fluorescence detection in AUC and highlight a few of its capabilities using fluorescent dye-labelled proteins and silica particles, graphene oxide as well as CdSe/ZnS quantum dots.

## Materials and methods

### Photoluminescence spectrometer

Measurements of the emission spectra of samples at ambient conditions were performed with a FluoroLog-3 photoluminescence spectrometer from Horiba.

### UV/Vis spectrometer

Extinction data was acquired with a Specord 210 Plus from Analytik Jena.

### MWE-AUC

An Optima XL-80K ultracentrifuge from Beckman Coulter was equipped with a custom-made fluorescence setup. The excitation wavelength was 518 nm. Details on the used parts can be found in the ESI.†

### MWL-AUC

For the MWL-AUC experiments, a user-modified preparative ultracentrifuge from Beckman Coulter (Optima L-90K) equipped with a multiwavelength extinction detector was used.^22^ For all experiments two-sector titanium centerpieces with an optical path length of 12 mm from Nanolytics were used. Temperature was kept constant at 20 °C.

### Graphene oxide

Graphene oxide was synthesized following the modified Hummers' method.^20,21^ Graphite was dispersed in concentrated sulfuric acid and potassium permanganate was slowly added under continuous stirring at temperatures below 10 °C. Further steps included workup with diluted sulfuric acid, followed by addition of water and diluted hydrogen peroxide. The as-synthesized graphene oxide was washed with water and delaminated using tip sonication. The dispersion was diluted to a concentration of 0.45 g L^−1^ prior to ultrasound processing with a UP-200S tip sonotrode from Hielscher Ultrasonics for 20 minutes (50% and duty cycle 0.5) at 10 °C. After processing, the sample was further diluted 1 : 1 with Millipore water. Prior to the measurement, the sample was processed using bath sonication for 10 minutes. The sample was measured in a constant radius experiment using a constant rotor speed of 10 000 rpm and a radial position of 6.8 cm.

### Silica particles

Fluorescent silica particles (50 nm sicastar®-redF with plain surface) from Micromod Partikeltechnologie were used for the experiments. Samples were diluted with Millipore water to 1 mg mL^−1^ (MWE-AUC) and 5 mg mL^−1^ (MWL-AUC). Prior to any measurement, samples were processed using bath sonication for 10 minutes. Samples were measured in a constant radius experiment using a constant rotor speed of 8000 rpm and a radial position of 6.8 cm.

### Fluorescent protein

A fluorescently labelled protein (Albumin–fluorescein isothiocyanate conjugate) from Sigma Aldrich was used for the sedimentation velocity experiments (40 000 rpm, 20 °C). 0.06 wt% of protein was dissolved without any purification in a Tris/NaCl buffer (12 and 15 mM, respectively).

### Quantum dots

CdSe/ZnS core–shell quantum dots stabilized with carboxylic acid in water (*λ*_em_ = 540 nm, 1 mg mL^−1^) were purchased from Sigma Aldrich and studied after 10 minutes of bath sonication. Samples were measured in a sedimentation velocity experiment using a rotor speed of 35 000 rpm at 20 °C.

### SEDFIT

SEDFIT^23^ version 16.1 was used to determine the continuous c(s) distributions. For the data evaluation, a resolution of at least 100 data points between 0.1 and 10 S for the protein and 200 data points between 1 and 30 S for the quantum dots were used. Radial- (RI) and time-invariant (TI) noise as well as the meniscus were fitted. For the fluorescence data, the signal was normalized to make it adjustable in SEDFIT using the fluorescence tools.^18^ Radial magnification gradient, temporal linear magnification drift, exponential photophysical intensity change, radial convolution from detection cone with excitation shadow and nonlinear detection were fitted in the first step together with RI and TI noise and the meniscus position. Then the fluorescence parameters were kept constant while RI and TI noise and the meniscus position were fitted again. SEDFIT standard parameters (*v̄* = 0.73 cm g^−1^, *ρ*_S_ = 1.0 g cm^−1^, *η* = 0.01002 poise) were used for the data evaluation, as the partial specific volume of the protein with attached dye is unknown. The confidence level of the regularization (max entropy for the protein and 2^nd^ derivative for the quantum dots) was set to 0.95.

### Constant radius experiments and data evaluation *via* HDR-MULTIFIT

Constant radius experiments, which are derived from gravitational sweep experiments^4,15,16^ allow the analysis of broad particle size distributions. This is achieved by constantly increasing the rotor speed, while evaluating the temporal evolution of the particle concentration at a constant radial position. In order to increase the signal-to-noise ratio of the detected spectra, several light flashes of low intensity can be accumulated on the detector's CCD chip prior to readout.^3,24^ This, however, restricts the experiment to constant rotor speeds, therefore throughout this manuscript it is called constant radius experiment. This combination of AUC techniques is especially useful for the accurate determination of optical spectra and sedimentation coefficient distributions with significant tailing for larger values. It was used for the evaluation of silica and graphene oxide particles.

Data was evaluated in HDR-MULTIFIT (available from J. Walter) using a direct boundary model^25^ with 150 grid points and a regularization parameter of 0.95, which provides non-normalized cumulative sedimentation coefficient distributions for every evaluated emission wavelength.

## Results and discussion

### AUC theory

The theoretical basics of AUC can be found in the literature.^26–28^ Briefly, the radial and temporal evolution of the particle concentration *c* is given by the Lamm equation^29^ with the radius *r*, the time *t*, the angular velocity *ω* and the sedimentation and diffusion coefficients *s* and *D*:1
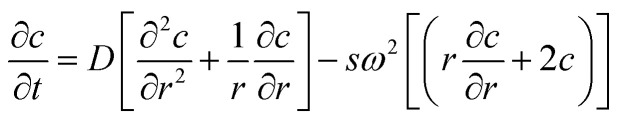
The sedimentation coefficient *s* is defined as the sedimentation velocity *u* normalized to the applied centrifugal field and can be expressed in terms of particle mass *m*, partial specific volume of the particle *v̄*, friction factor *f* and solvent density *ρ*_S_:2
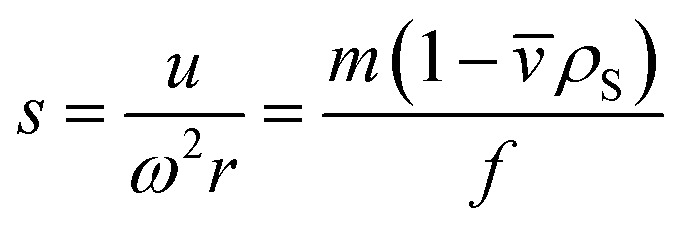
In general, the sedimentation coefficient *s* depends on the size and the shape of the particles under consideration as well as on their density. For spherical particles eqn (2) can be simplified, as the hydrodynamic diameter *x*_H_ within the friction factor *f* = 3π*ηx*_H_ matches the geometric particle diameter *d*. Therefore, the latter can be calculated from the measured sedimentation coefficient with the solvent viscosity *η*:3
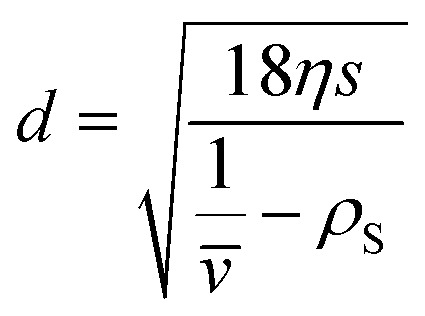


The frictional properties also influence the diffusion coefficient *D*:4
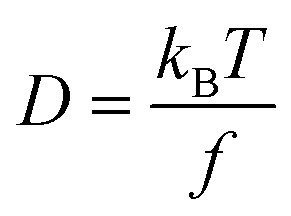
The diffusion coefficient thus only depends on the friction factor *f*, which is determined by the size and shape of the particle, as well as on the thermal energy represented by the Boltzmann constant *k*_B_ and the temperature *T*. As both the sedimentation and the diffusion coefficient depend on the friction factor *f*, the combined knowledge of *s* and *D* can be used to derive the molar mass of the particle *via* the Svedberg equation.^30^ In terms of data analysis, it is convenient to describe the diffusion coefficient *D* as a function of the sedimentation coefficient *s* and the frictional ratio *f*/*f*_0_, which is the ratio of the hydrodynamic to the volume equivalent diameter. The two equivalent diameters describe the size of a spherical particle having the same properties (volume or friction, respectively) as the analyzed anisotropic particle. Within the c(s) analysis method in SEDFIT,^23^ it is possible to vary *f*/*f*_0_ or *D*, which can be used interchangeably for a single species:5
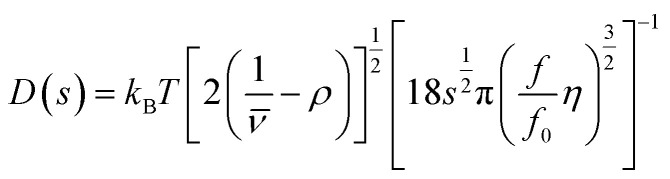


### Data acquisition and evaluation

**Fig. 1:**
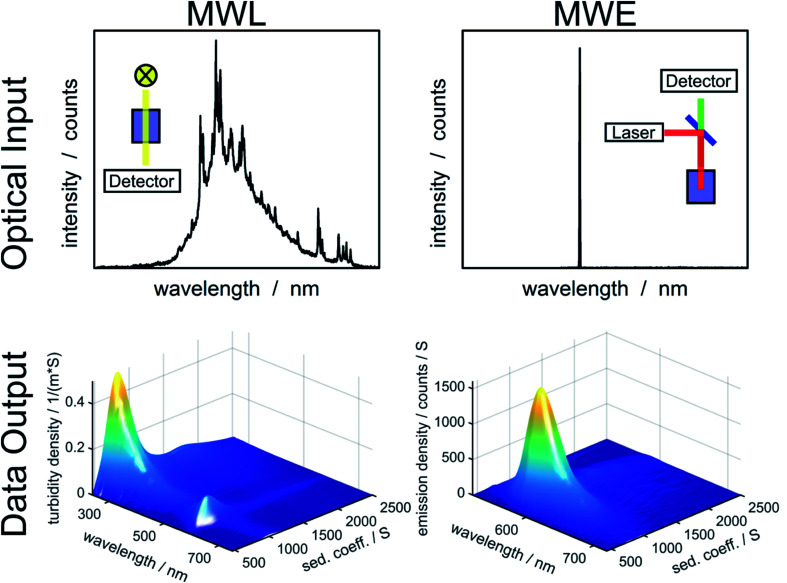
Overview of optical input to the MWL- and MWE-AUC setups and data output from the analysis of an experiment with fluorescent labelled silica particles using HDR-MULTIFIT.^4^

In order to determine diffusion coefficient (eqn (5)), sedimentation coefficient (eqn (2)) or particle size (eqn (3)) distributions, the measured sedimentation boundaries need to be analyzed using numerical solutions to the Lamm equation (eqn (1)). The integral sedimentation boundary results from a superposition of the sedimentation boundaries of individual species. The concentration of each fraction must be correctly represented within the distribution. Although concentration is not directly observable for MWE- and MWL-AUC, it can be calculated from the radial and temporal evolution of the acquired optical signal. Data from MWL-AUC, for instance, can be analyzed using the extinction coefficient, which depends on the complex refractive index as well as on particle size and shape. However, even without this coefficient, the individual fractions can be weighted according to their optical signal, which allows determining extinction or emission weighted sedimentation coefficient distributions for all available wavelengths. *Vice versa*, emission or extinction spectra can be extracted for individual species as can be seen in Fig. 1.

Additionally to varying weighting due to different optical signals, the concentration sensitivities of MWL- and MWE-AUC are different. MWL-AUC has the advantage of a free choice of wavelength in a broad spectral range, which allows obtaining data of good quality in the majority of studies. This is more complicated in the case of MWE-AUC because the optical response for MWE-AUC depends heavily on the optical properties of the sample in proximity of the excitation wavelength (see Fig. 1). Especially, the inner filter effect can lead to a non-linear relation between emission signal and concentration. This happens because the sample extinction decreases the excitation intensity on the way to the light focus in the sample as well as the intensity of the emitted light on the way to the detector. The inner filter effect can be avoided by using low concentrations. However, in order to obtain sufficient signal from weakly emitting samples, strong excitation may be required, which can lead to photodegradation and an distortion of the sedimentation coefficient distribution.

### Optics and movement of excitation source

**Fig. 2:**
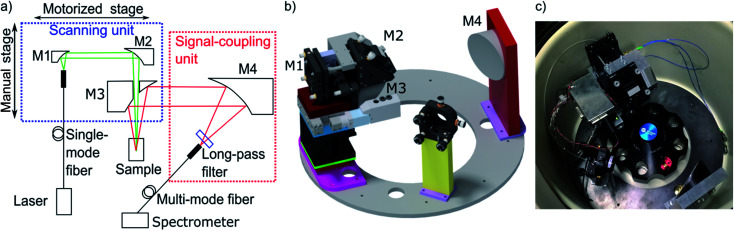
(a) Main components of the optical setup projected in a single plane. (b) Three-dimensional depiction of setup (fibers are not shown). Optics and optomechanics are depicted with permission from Thorlabs^32^ and Newport,^33^ the linear actuator T-NA-SV2 is shown with permission from Zaber^34^ (http://www.zaber.com). (c) Photograph of the setup implemented in the vacuum chamber of the XL-80K centrifuge with fiber optics and electronics distributor box.

The MWE-AUC design, as shown in Fig. 2, is based partly on concepts used for MWL-AUC^31^ and the established confocal fluorescence detector.^12^ There are two main setup parts: the scanning unit and the signal-coupling unit. The scanning unit is responsible for providing the focused laser light at the correct radial position in the sample cell and collecting the signal emitted by the probe. At the same time it is able to perform a radial scan *via* a step motor, which is mounted on a linear translation stage. The signal-coupling unit focuses the collimated signal beam into a multimode glass fiber forwarding it to the spectrometer. A detailed list of components can be found in the ESI.†

The excitation light from the pump laser is coupled in a single-mode fiber and then fed into the vacuum chamber *via* a vacuum feedthrough. Using a laser diode allows direct amplitude modulation of the laser power *via* a TTL pulse from a timer card. Additionally, the laser pulse intensity can be adjusted by attenuating the amplitude of the TTL pulse *via* a simple potential divider based on a multi-turn potentiometer.

In the scanning unit (see Fig. 2), the laser light from the single-mode fiber is collimated with an off-axis parabolic (OAP) mirror M1 and is focused in the measurement cell *via* the OAP mirror M2 after passing through the central hole of OAP mirror M3. The light, emitted and backscattered (fluorescence, scattering and reflection) from the sample is collected and collimated by OAP mirror M3 and then focused by the OAP mirror M4 in a multi-mode step-index fiber after traveling through a spectral longpass filter. After the fiber vacuum feedthrough, the light is coupled into the spectrometer. Change of the scanning-unit position on the radial axis has no effect on light propagation because the beam is collimated. Furthermore, mirror M4 is larger than M3 to allow for different height adjustments of the scanning unit without changing the efficiency of light coupling into the multi-mode fiber. There are several advantages of using OPA mirrors instead of lenses for the fluorescence setup as used in the commercial fluorescence detector.^28^ The major benefit is the elimination of chromatic and spherical aberrations as it was already shown for the MWL-AUC.^24^ Additionally, the signal quality is improved due to the reduction of the fluorescence background.^35^ A drawback of the protected silver OAP mirrors is the decreasing reflectance below 90% for wavelengths lower than 400 nm.^32^ With four OAP mirrors being used in the setup, the signal already decreases by 35%. Changing to UV-enhanced aluminium OAP mirrors would allow covering also the UV range down to 250 nm. However, in comparison to protected silver OAP mirrors, the reflectance in the visible range is reduced. The spectral experimental range is therefore currently limited by the optics to wavelengths above 400 nm, while the upper wavelength limit is determined by the spectrometer at 1100 nm. With different fibers, laser sources and spectrometer the range could be extended up to about 2500 nm.

### Mechanical construction

An Optima XL-80K ultracentrifuge from Beckman Coulter was modified to provide the possibility of fluorescence spectrum detection. Holes for the vacuum feedthroughs – for the glass fibers, for the electronics for the light barrier for angular position detection and for the step motor – were drilled in the heatsink. The scanning unit and the signal-coupling unit are both mounted on a laser-cut steel ring to facilitate accurate alignment of all components. The steel ring has an inner diameter of 192 mm, a bit larger than the rotor, which means that it does not have to be placed below the rotor, thus avoiding any potential accident with an imbalanced rotor. The ring is mounted on three spacers, which themselves are screwed to the vacuum chamber. While mirror M4 is fixed in position and alignment, all other components are at least partly adjustable. The main part of the scanning unit is mounted on a cage system. The glass fiber delivering the laser light is directly mounted on a bracket, which is attached to a cage rod, shared by mirrors M1 and M2 and a lower cage rod, connected to mirror M1 only. Due to the restricted space and the holder of mirror M3, only the upper cage rods are used to connect the holders of mirrors M1 and M2. The connection of M3 with M1 and M2 is achieved by a custom aluminium part (see ESI†) that also allows stabilizing M2 against tilting during the optical adjustment. To perform a radial scan, all the mirrors of the scanning unit – M1, M2, and M3 – are mounted on a linear translation stage with a step motor. This linear stage itself is mounted on a linear z-stage with manual translation, which enables to adjust the height of the scanning unit and therefore the focus position of the laser beam and, correspondingly, the detection volume in the sample. Mirror M4 is fixed on the steel ring, while the multi-mode fiber, which guides the detected light to the spectrometer, is attached to a 5-axis mount.

### Alignment procedure

As the setup consists of two practically independent optical paths – the pump laser beam and that of emitted and back-scattered light – at first each of them has to be adjusted individually. The mandatory superposition of both beams in the sample cell is to be provided at the end. The mirror M1 and the fiber tip in the FC/APC connector with tuneable focal position are adjusted to provide a circular collimated laser beam, which hits the middle of the mirror M2. The position of mirror M2 on the cage system is optimized to center the pump beam in the hole of mirror M3. Further adjustments are necessary to provide positioning of the excitation laser beam waist in the common focal plane of the mirrors M2 and M3 in the sample cell and to ensure the alignment of the beam straight in vertical direction.

An additional laser pointer, coupled to a single-mode fiber, can be used to invert the path of the scattered light for the adjustment of the 5-axis mount in the signal-coupling unit, as well as of the mirror M3 in the scanning unit for the best possible overlap of the laser beams over the common path in the sample cell. First, the 5-axis mount is adjusted in such a way that the laser beam hits the middle of mirror M4 by tilting the fiber tip. In a later step, the shape of the inverted laser spot at the sample position is made spherical by turning and tilting M3. Care should be taken that the optical axis of the inverted laser is also vertically aligned at the sample position. During alignment some turning of the mirrors might be necessary to provide a circular shape of the laser spots at the sample position. Finally, the two laser spots are brought together vertically and radially by fine adjusting the *x*–*y* position of the fiber tip of the inverted laser as well as the tilting angle of mirror M2.

### Calibration

AUC is a material-standard-free measurement technique. However, systematic errors may emerge from incorrect radial and angular positioning or temperature inaccuracy.^36^ Therefore, these parameters need to be carefully calibrated. Synchronization of spectrometer triggering and laser modulation with rotor rotation is achieved *via* a light barrier, triggering electronics and an adapted LabVIEW©-based data acquisition software developed previously.^22^ Details on the AUC triggering have been recently published elsewhere.^31^ In short, the temporal delay between the detection of a polished area on the rotor bottom *via* a light barrier and the sample cell arriving at the measurement position is a function of rotor speed and has to be calibrated using a fluorescent dye solution as a test sample.

Similarly, the radial position of the motorized stage needs to be evaluated. Therefore, radial calibration is performed with the counterbalance from Beckman Coulter. The counterbalance is installed in the rotor upside down and the reflection of the excitation laser light from the edges of the inner and outer mask is recorded at zero speed to avoid rotor stretching and loosening of the inverted weight. Turning the counterbalance is necessary to detect the reflection. More details on the radial and angular calibration can be found in the ESI.†

Due to the several additional metal parts being located within the vacuum chamber, temperature control measurements at the top of the counterbalance at zero rotational speed were performed.^37^ The temperature control of the centrifuge shows a temperature offset of +2 °C, which is taken into account within the software, the experiment and the data evaluation. The deviation was already present prior to any modification of the centrifuge and can therefore be attributed to the centrifuge and not to the setup. Details on the temperature measurements can be found in the ESI.†

### Validation of fluorescence signal

**Fig. 3:**
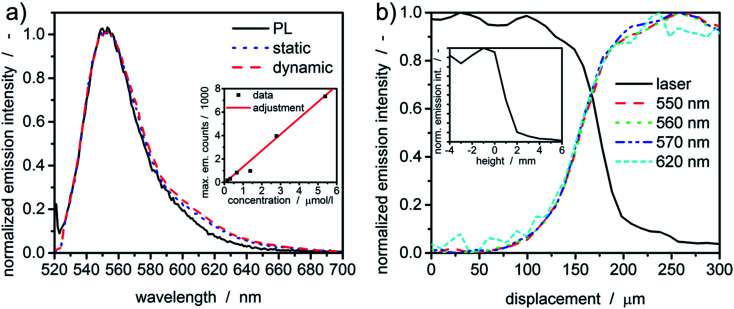
(a) Normalized emission spectra of rhodamine 6G acquired with MWE-AUC under static conditions and within a constant radius experiment (dynamic) as well as obtained with the benchtop photoluminescence spectrometer (PL). The inset shows the linear correlation between fluorescence signal and the concentration of fluorescent dye. The data point at ≈1.4 μmol L^−1^ was excluded from the adjustment. (b) Normalized intensity signal for a single radial scan of a liquid edge of dissolved rhodamine 6G at 10 000 rpm. The inset shows the emission signal as a function of the focus height position within the sample under static conditions.

Prior to any measurement, the spectral sensitivity of the new setup was evaluated. Therefore, the fluorescent dye rhodamine 6G was dissolved in water and its fluorescence spectra were measured under static and dynamic rotor conditions within the MWE-AUC and in the benchtop photoluminescence spectrometer. As one can see in Fig. 3, spectra obtained with the MWE-AUC setup under static and dynamic conditions, are very similar and match also with the data obtained by the photoluminescence spectrometer. Small deviations in the red wing can be explained in terms of the spectral properties of optical components used, which exhibit some wavelength dependence of transmission and reflectance parameters.

Additionally, a concentration series of rhodamine 6G was used to assure the signal linearity of the acquired emission intensity. Measured extinction spectra enable to calculate the concentration of the fluorescent dye^38^ in water and to correlate it with the maximum emission intensity as described in the ESI.† Overall, a linear correlation for moderate concentrations (≈μM) could be found (Fig. 3(a)), which allows correlating the emission intensity directly with the dye concentration.

### Confocal characteristics

Radial and axial resolutions are important for signal generation and data evaluation. The meniscus (“liquid edge”) of a fluorescent dye solution in water can be used to estimate the radial resolution of the setup.^24^ In comparison to the cell bottom or the counterbalance, it gives larger values for the radial resolution for MWL-AUC.^24^ The radial intensity distributions for the excitation and a few emission wavelengths are given in Fig. 3(b). Notably, the intensity of the laser light (518 nm) reflected from the bottom surface of the upper cell window, which changes due to the change in refractive index at the gas-water-glass interface, gives a similar indication of the meniscus position as the fluorescence signal. Additionally, the fluorescence intensity distribution is similar for different wavelengths as a consequence of the used chromatic aberration-free parabolic mirrors. The distribution width between 10% and 90% intensity is approximately 100 μm.

The same sample can also be used to characterize the axial confocal parameter in static mode. Therefore, the height of the scanning unit was adjusted *via* the linear z-stage and the mean emission intensity (522–700 nm) was recorded. From the results in Fig. 3, the axial resolution can be approximated to be ≈2 mm.

### Sedimentation velocity experiment

**Fig. 4:**
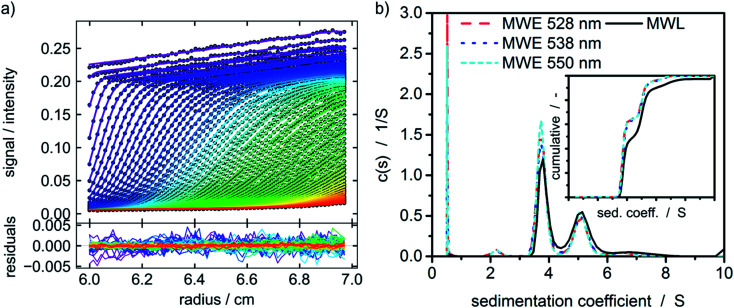
(a) Raw data of the MWE-AUC experiment of the albumin-fluorescein isothiocyanate conjugate at 550 nm and a rotor speed of 40 000 rpm with model curves from the c(s) method implemented in SEDFIT.^18,41^ (b) Comparison of c(s) distribution obtained *via* MWL-AUC at 495 nm (*f*/*f*_0_ = 1.67) and MWE-AUC (*f*/*f*_0_ = 1.66, 1.66, and 1.64) for different wavelengths from the analysis in SEDFIT. The inset shows the cumulative sedimentation coefficient distributions neglecting species below 3 S.

A protein with attached fluorescence dye was used to characterize the performance of the MWE-AUC setup. The acquired data was analyzed with SEDFIT using the built-in fluorescence tools^18^ (see Fig. 4). The measured data is very similar to the already reported datasets of fluorescence without spectral discrimination,^18^ including an increase of signal magnitude with radius. This effect is considered to originate from a shift of the focal point during the radial movement of the detector setup^18,39^ but can be well accounted for in SEDFIT. The major improvement in comparison to the established fluorescence AUC is the now available spectral data. The resulting sedimentation coefficient distributions in Fig. 4 and the fitted frictional ratios (*f*/*f*_0_ = 1.66, 1.66, and 1.64 for 528 nm, 538 nm and 550 nm, accordingly) are very similar for different evaluation wavelengths. This means that as expected for uniform samples, sedimentation and diffusion properties can be analyzed equally well for different wavelengths of the fluorescence spectrum. This is the prerequisite for the simultaneous hydrodynamic, thermodynamic and spectral characterization of macromolecules and nanoparticles within the MWE-AUC. A depiction of the corresponding raw data and an exemplary spectrum can be found in the ESI.† In order to fully validate the setup and due to the fact that the fluorescent dye might change the hydrodynamic and thermodynamic properties of the protein, the sample was also studied with the MWL-AUC setup. The comparison of the results obtained from MWE-AUC for different wavelengths and the MWL-AUC setup is depicted in Fig. 4. The position of the three main species and the fitted frictional ratio (*f*/*f*_0_ = 1.67 for MWL-AUC) can be well retrieved, meaning that sedimentation and diffusion coefficients are in excellent agreement between MWE-AUC and MWL-AUC. Slight differences occur for species below 3S. Most likely, these correspond to the fluorescent dye that desorbed from the protein, either at the preparation stage or during the experiment. As the same sample was investigated, both setups should deliver similar results for the relative concentration of the free dye. However, in the MWL-AUC, the relative dye concentration amounts to less than 1%, while the MWE-AUC gives values up to 8%. It is well known that results from different AUC detection systems may indeed be slightly altered.^36^ There are several reasons to explain that. First of all, the different detection systems exhibit different concentration ranges that appear convenient for thorough analysis. Secondly, distributions generally depend on the measurement technique used, as fractions of particles may contribute differently to the overall acquired signal for different detectors.^40^ Thus, due to the different emission properties of covalently bound and free fluorophores, the relative signal intensities can be altered. Another difference between MWE- and MWL-AUC is photodegradation that is present only within the MWE-AUC. Due to the small size of the free fluorescent dye, it does not show significant sedimentation at the applied rotor speed but is constantly subjected to excitation. Therefore, a decrease of emission due to photodegradation could be misinterpreted as sedimenting species.

### Constant radius experiment

In order to test the performance of the setup in terms of spectral sensitivity and higher values of sedimentation coefficients, fluorescent silica particles were characterized using a constant radius experiment.^4,15,16^ The constant radius experiment is chosen as it allows an easier access to the spectral properties of a sample. Moreover, the radial characteristics of the fluorescent setup, as shown in Fig. 4, do not play a significant role due to the static measurement position. Fluorescent silica particles were measured both with MWL-AUC and MWE-AUC. In order to avoid any deviation of sedimentation coefficient distributions due to the size-dependent scattering of the silica particles, the evaluation of the sedimentation data from MWL-AUC was undertaken at the extinction peak of the fluorescent dye. However, due to the boundary conditions of the two setups, different concentrations (1 mg mL^−1^ in MWE- and 5 mg mL^−1^ in MWL-AUC) had to be investigated. Otherwise, the optical signal of the MWE-AUC would be compromised by the inner filter effect or the MWL-AUC would not be able to measure sufficiently high extinction signals. The free fluorescent dye, which does not show meniscus depletion, can be used after the measurement to detect the radial position of the meniscus, which is necessary for data evaluation.

**Fig. 5:**
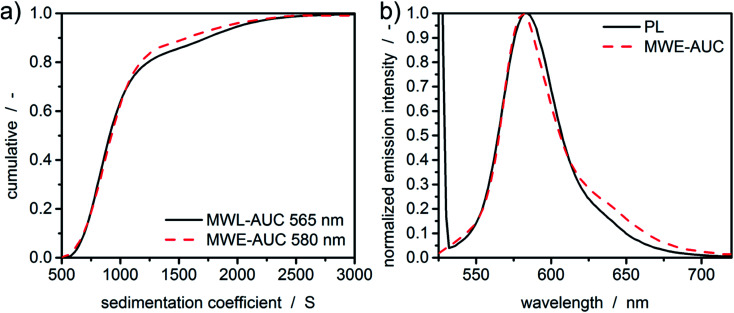
(a) Sedimentation coefficient distributions for the fluorescent silica particles obtained by MWL-AUC and MWE-AUC *via* HDR-MULTIFIT. (b) Emission spectra extracted from the constant radius experiment for the entire sedimentation coefficient distribution compared to the results from the benchtop photoluminescence spectrometer (PL).

Results for the sedimentation coefficient as well as the spectra extracted from HDR-MULTIFIT^4^ can be found in Fig. 5. The excellent agreement of the sedimentation coefficient distributions of MWL-AUC and MWE-AUC shows that the new device is working properly and provides accurate results not only in terms of sedimentation coefficients but also in terms of the detected concentration ratios. Additionally, it can be seen that the spectra extracted from the sedimentation coefficient distributions are matching the spectra obtained by the benchtop photoluminescence spectrometer, showing only a minor deviation on the red spectrum side, similar to Fig. 3.

### Fluorescence emission of graphene oxide and quantum dots

So far, the acquired fluorescence spectra originated from the fluorescent dyes. Particularly interesting is the analysis of particulate samples that show intrinsic fluorescence. To illustrate this, an ultrasound-processed sample of graphene oxide was investigated in a constant radius experiment. Graphene as a zero-bandgap semiconductive material does not display any photoluminescence. However, introducing functional groups such as hydroxy groups or epoxides opens the bandgap, giving new routes for determining its structure using fluorescence measurements.

**Fig. 6:**
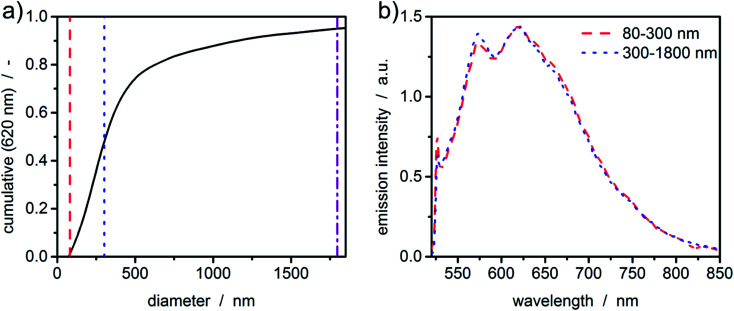
Results of the constant radius experiment for the processed graphene oxide sample obtained *via* HDR-MULTIFIT. (a) Cumulative diameter distribution obtained from the sedimentation coefficient distribution at 620 nm. (b) Extracted spectra of the graphene oxide platelets. Colours and dashed and dotted lines are corresponding to the lower boundaries of the respective size interval in (a).

With the graphene oxide being a monolayer material, the sedimentation velocity is directly related to the lateral dimension of the platelet. Therefore, sedimentation coefficients can be directly transformed to equivalent diameters using the method and parameters published in literature.^6^ As can be seen in Fig. 6(a), even broad size distributions with sizes up to the micrometer range can be measured in accordance with typical samples originating from this synthetic route.^42^ The fluorescence spectrum of graphene oxide shows typical green emission in the range of 500-700 nm.^43,44^ Graphene oxide is a network of non-uniform regions with sp^2^- and sp^3^-hybridized carbon atoms. In such materials, a mixture of sp^2^- and sp^3^-bondings determines the opto-electronic properties by the π states of the sp^2^-sites. The green to infrared emission region (500–800 nm) as shown in Fig. 6(b) is suggested to originate from localized electronic structure, neighbouring functional groups or can refer to sp^2^ carbon regions captured by repulsive sp^3^ carbon hard wall barriers. Further, the bond alteration inducing intervalley scattering effects can also cause the emission.^45^ The intensity of the photoluminescence correlates with the degree of defects in the carbon framework.

Although the sample is polydisperse, hardly any deviations of the fluorescence signal for the two intervals can be observed. This leads to the conclusion that the sample is quite uniform in terms of the chemical composition.

**Fig. 7:**
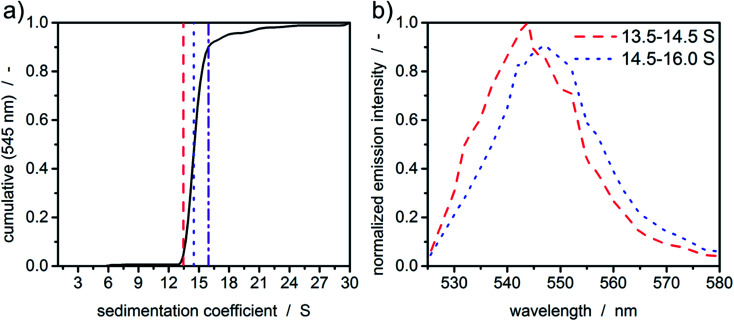
(a) Cumulative c(s) distribution of CdSe/ZnS quantum dots at 545 nm and sedimentation coefficient intervals used for the extraction of fluorescence spectra. (b) Extracted normalized spectra of the quantum dots. Colour and dashed and dotted lines are similar to the lower boundary of the respective interval in (a).

In order to demonstrate the potential of MWE-AUC with respect to the analysis of size- and shape-dependent emission properties of nanoparticles, we show in Fig. 7 exemplary results for CdSe/ZnS quantum dots, where the emission intensity as well as the spectral peak position depends on the CdSe core size as well as on the thickness of the ZnS shell.^46^ As shown in Fig. 7(a), the cumulative sedimentation coefficient distribution is very narrow, therefore only two intervals with equal increment between 0.05 and 0.9 of the cumulative distribution are used for the extraction of spectra. The values for the interval boundaries were chosen to exclude small side products and agglomerates based on the shape of the sedimentation coefficient distribution. Fig. 7(b) gives a clear indication of a sedimentation coefficient dependent spectral shift of only 3 nm, which demonstrates the ability of the MWE-AUC setup. The red shift can be explained with larger core sizes and/or shell thickness, which is plausible to be correlated with higher sedimentation coefficients. On this basis, complex structure–property relationships can be studied now in unprecedented detail *via* MWE-AUC.

## Conclusion

A new multiwavelength emission detector for analytical ultracentrifugation (MWE-AUC) was developed and is presented in this paper. The analysis of fluorescent-labelled proteins and silica particles, graphene oxide and CdSe/ZnS quantum dots demonstrated the capability of hydrodynamic and optical characterization of nanoparticles and macromolecules using the new MWE-AUC setup. Hence, new and so far inaccessible applications could be the stoichiometric study of multi-protein complexes and their binding behaviour, the identification of different species in carbon allotropes such as carbon nanodots or graphene quantum dots. In particular, the fluorescence spectra can provide details on size- and structure-dependent emission characteristics of quantum materials as it has been shown for the exemplary case of core–shell CdSe/ZnS quantum dots. Now a comprehensive toolbox for the optical characterization of nanoparticles by combined analysis of size- and shape-dependent extinction (MWL-AUC) and fluorescence (MWE-AUC) properties is available.

## Conflicts of interest

There are no conflicts to declare.

## Supplementary Material

NA-001-C9NA00487D-s001
