# Supplementary material for: A multiwavelength emission detector for analytical ultracentrifugation
Source: Nanoscale Adv. 2019 Oct 4;1(11):4422–32. doi: 10.1039/c9na00487d (PMC9419176; doi:10.1039/c9na00487d)
Supplement: NA-001-C9NA00487D-s001 [file NA-001-C9NA00487D-s001.pdf]

Electronic Supplementary Material (ESI) for Nanoscale Advances.
This journal is © The Royal Society of Chemistry 2019

Electronic Supplementary Information

A multiwavelength emission detector for analytical ultracentrifugation

Simon E. Wawra, Georgy Onishchukov, Maria Maranska, Siegfried Eigler, Johannes Walter, Wolfgang Peukert

Optical components

Identifier	Description	Supplier	Model
M1	1/2" 90° off-axis parabolic mirror, prot. silver coating, RFL = 15 mm	Thorlabs	MPD00M9-P01
M2	Ø1" 90° off-axis parabolic mirror, prot. silver coating, RFL = 6"	Thorlabs	MPD169-P01
M3	Ø2" 90° off-axis parabolic mirror with Ø3 mm hole, prot. silver coating, RFL = 4"	Thorlabs	MPD249H-P01
M4	Ø3" 45° off-axis parabolic mirror, prot. silver coating, RFL = 6"	Thorlabs	MPD364-P01
Spectral filter	1" longpass filter, 520 nm	Omega	520AFLP
Laser	Diode laser with temperature control	Lasertack	PD-01298

Mechanical components

Identifier	Description	Supplier	Model
Fiber adapter	1/2" FC/APC fiber adapter plate	Thorlabs	SM05FCA
Fiber cage adapter	30 mm to 16 mm cage system right-angle adapter	Thorlabs	SP30
M1 mount	1" kinematic cage-compatible mount	Thorlabs	KC1/M
M1 adapter	1" adapter for Ø1/2" off-axis parabolic mirrors	Thorlabs	MP127P1
M2 mount	Right-angle kinematic OAP mirror mount for 30mm-cage system	Thorlabs	KCB1P/M
M3 mount	Ø2" precision kinematic mirror mount	Thorlabs	KS2
Fiber mount	1" 5-axis kinematic mount	Thorlabs	K5X1
Fiber adapter	FC/PC fiber adapter plate	Thorlabs	SM1FC
Cage rods	Cage assembly rod	Thorlabs	ER6
Linear stage	TravelMax stage	Thorlabs	LNR25M/M
Vertical linear stage	Vertical linear stage, dovetail, 14 mm travel	Newport	M-DS65-Z
Step motor	Low-vacuum micro linear actuator with built-in controller	Zaber	T-NA-SV2

Vacuum components

Identifier	Description	Supplier	Model
Fiber feedthrough	Single-mode QS-488 fiber (3.5/125/900 µm) with adjustable FC/APC connectors and multimode QM-UVVIS fiber (50/125/900 µm, NA 0.22)	OZ Optics	
Step-motor cable feedthrough	18 wires	Lemo	SWH.3S.318.CLLDV
Light-barrier cable feedthrough	4 wires	Lemo	EVP.0V.304.CLLSV

Electronic components

Identifier	Description	Supplier	Model
Light barrier	Reflective photosensor		RPR-220
NI card	Multifunctional analog/digital I/O card (3I/O counters needed)	National Instruments	NI PCIe-6321
Miniature grating spectrometer with CCD sensor		Ocean Optics	Flame-S

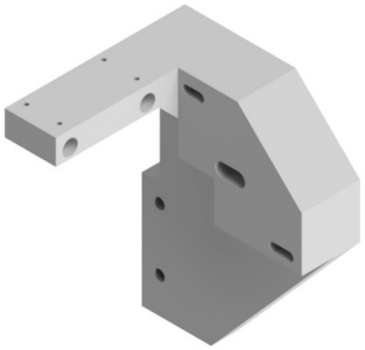

Figure S1 Custom-made aluminium attached to the mirror adapter of M3 and holding the cage rods, which connect the mirror adapters of mirrors M1 and M2.

Angular and radial calibration

It is convenient to express the rotation-speed dependence of the temporal delay between the detection of a polished area on the rotor bottom via a light barrier and the sample-cell arrival at the measurement position rather in terms of angular position of the sample. Therefore, this “angular position” of the sample cell is recorded for a number of rotation speed values. Angular calibration is performed via a mono-sector (schlieren) centerpiece filled with fluorescent rhodamine 6G in water. Starting at 1000 rpm, the speed is increased in steps of 1000 rpm up to the maximum speed of the An-50Ti rotor, while angular intensity

measurements are performed in the middle (in terms of radius) of the measurement cell. The number of accumulations is successively increased in order to obtain sufficient signal (> 3000 counts). The pulse width is set to 6°, 0.5µs are additionally added to take into account the delay of the laser-diode pulse. The measured angular intensity distributions are used to find the sample angular position as the angle of maximum intensity or the middle of the intensity plateau.

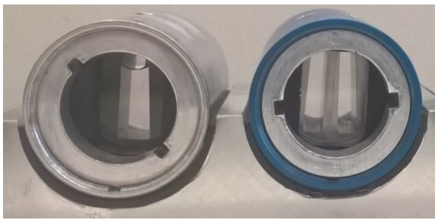

Figure S2 Comparison of mono-sector measurement cell for MWE-AUC and double-sector cell used in MWL-AUC.

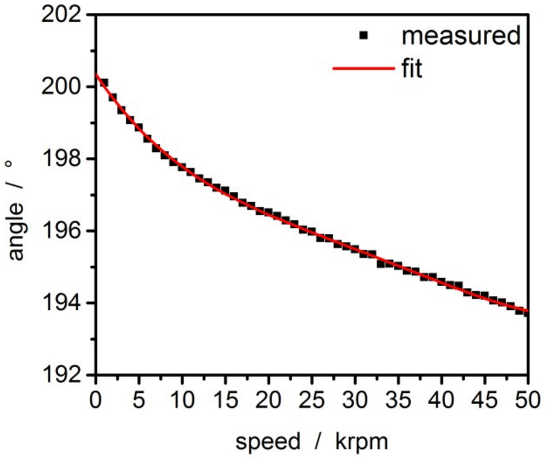

Figure S3 Results of angular calibration of the fluorescence setup and the fitted fourth-order polynomial.

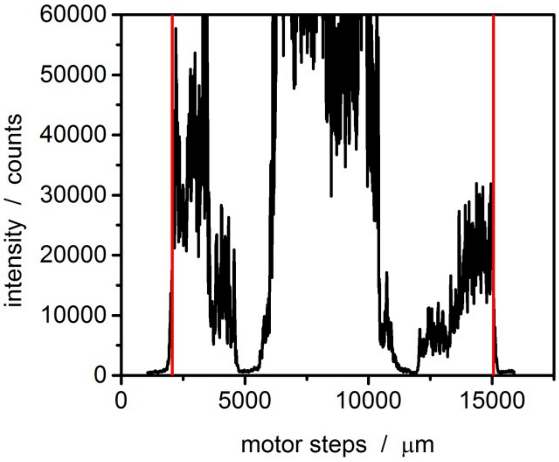

Figure S4 Radial intensity distribution of the laser light reflected from the counterbalance.

Temperature

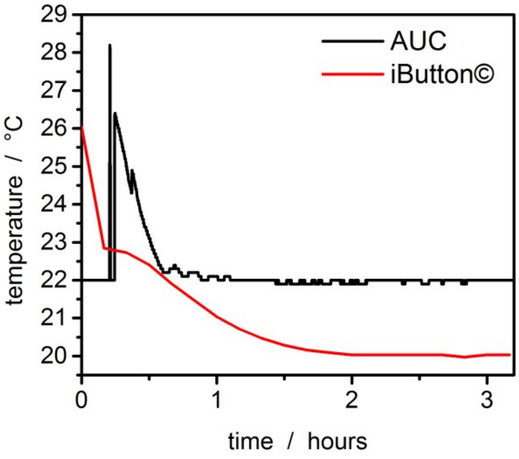

Figure S5 Evolution of temperature at the top of the counterbalance in the An-50Ti rotor within the vacuum chamber. The temperature at the AUC was set to 22°C.

Signal linearity

As the height of the setup was readjusted for every sample, the measurement was performed under static conditions in regular two-sector centerpiece measurement cells. The concentration was determined via extinction measurements.

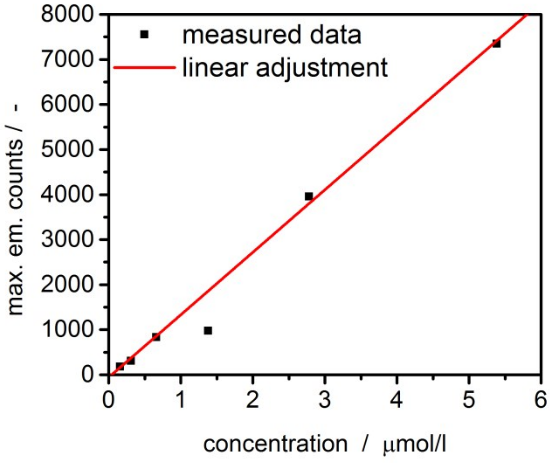

Figure S6 Maximum emission intensity of rhodamine 6G samples with different concentrations.

Sedimentation velocity experiments

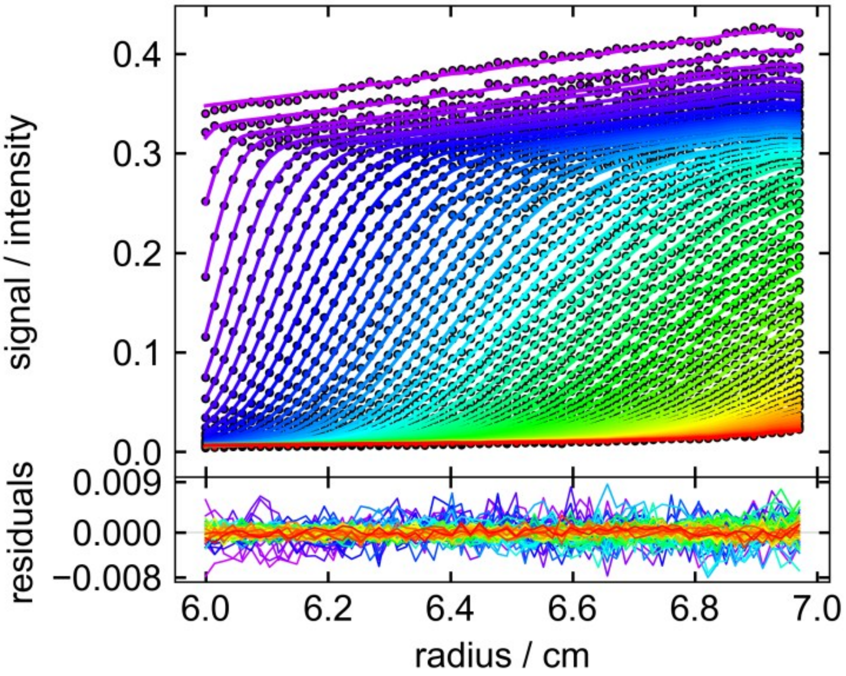

Figure S7 Raw data of MWE-AUC experiments of the albumin-fluorescein isothiocyanate conjugate at 538 nm and a rotor speed of 40 000 rpm with model curves from the c(s) method implemented in Sedfit.[1,2]

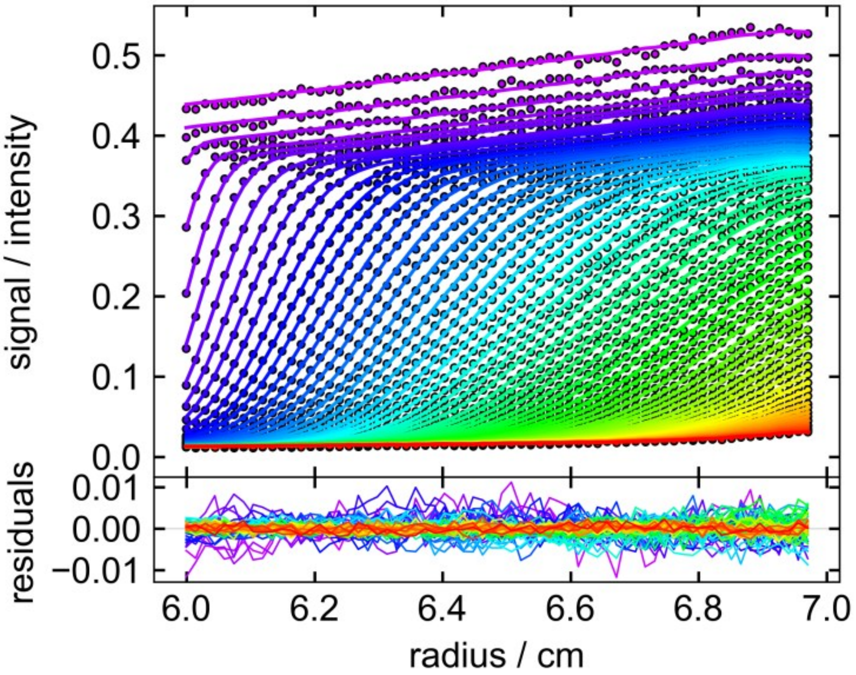

Figure S8 Raw data of MWE-AUC experiments of the albumin-fluorescein isothiocyanate conjugate at 528 nm and a rotor speed of 40 000 rpm with model curves from the c(s) method implemented in Sedfit.[1,2]

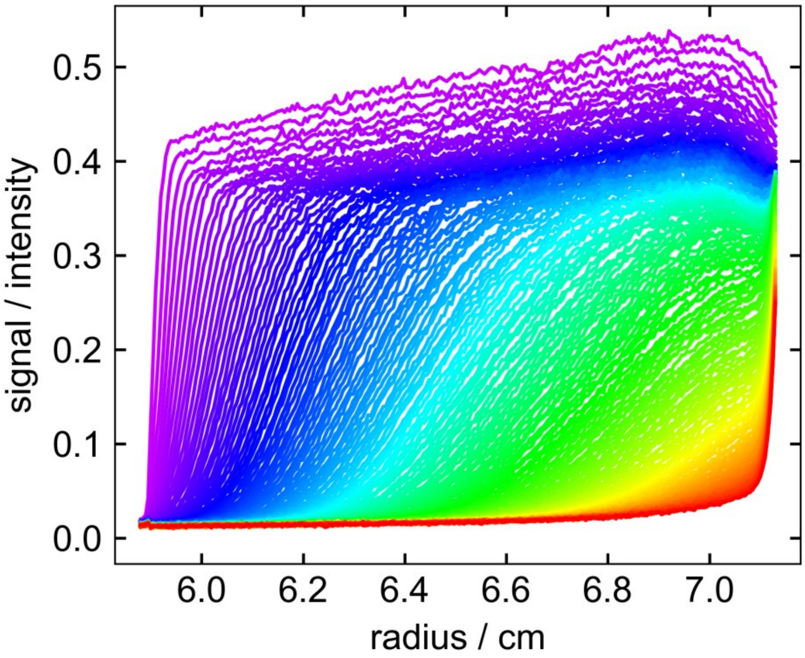

Figure S9 Raw data of MWE-AUC experiments of the albumin-fluorescein isothiocyanate conjugate at 528 nm and a rotor speed of 40 000 rpm including the complete radial range and every 2nd scan[2].

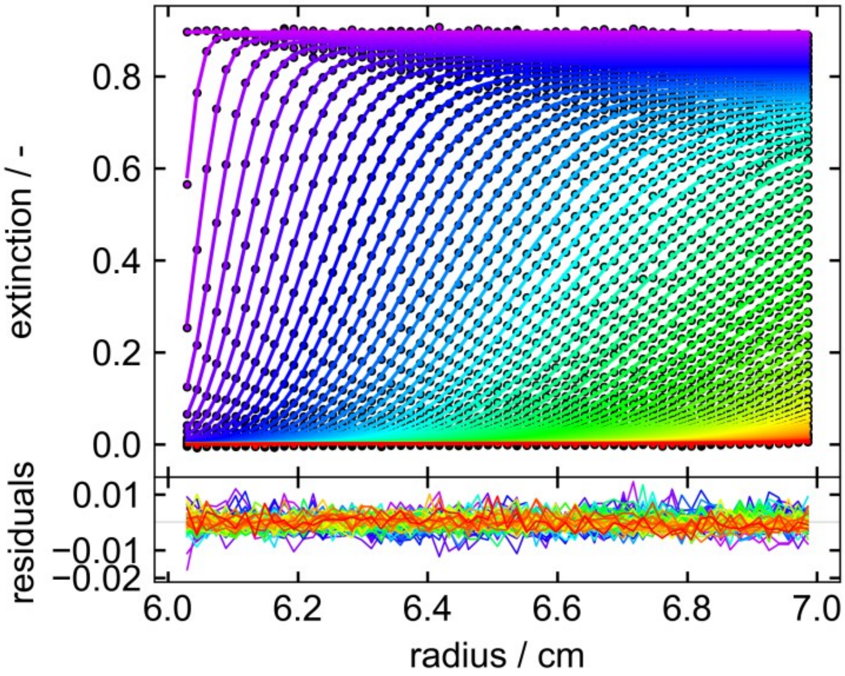

Figure S10 Raw data of MWL-AUC experiments of the albumin-fluorescein isothiocyanate conjugate at 495 nm and a rotor speed of 40 000 rpm with model curves from the c(s) method implemented in Sedfit.[2,3]

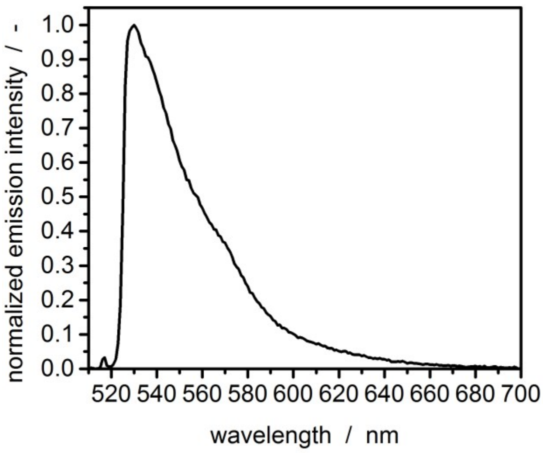

Figure S11 Exemplary normalized spectrum (scan 2) of albumin-fluorescein isothiocyanate conjugate measured at a radial position of 6.5 cm. The small peak at 518 nm corresponds to the residual pump.

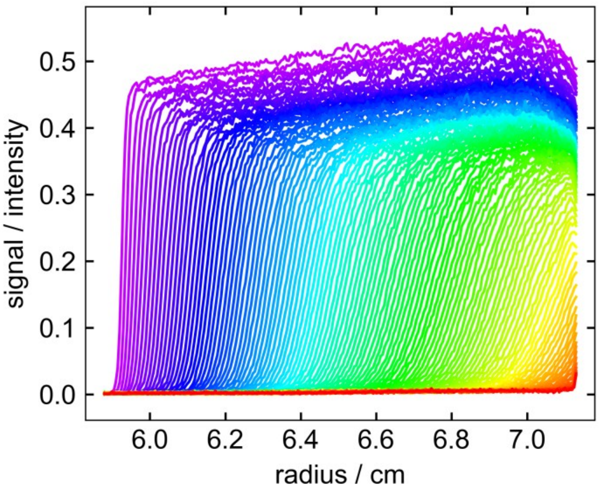

Figure S12 Raw data of MWE-AUC experiment of the CdSe/ZnS quantum dots at 545 nm and a rotor speed of 35 000 rpm including the complete radial range and every scan[2].

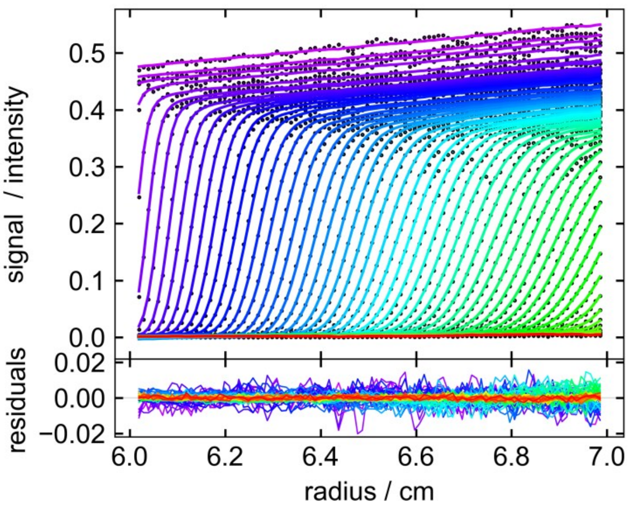

Figure S13 Raw data and fitted c(s) model curves of the MWE-AUC experiment of the CdSe/ZnS quantum dots at 545 nm and a rotor speed of 35 000 rpm including every second scan and data point[2].

Constant radius experiments

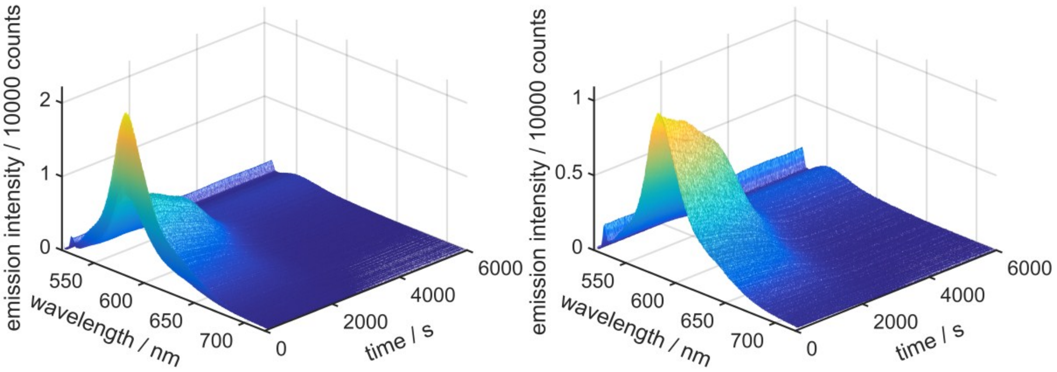

Figure S14 Raw data of the constant radius experiments for the silica particles. Please note that the laser intensity was readjusted at the beginning of the experiment. Therefore, the initial decrease of intensity is excluded from the analysis, as shown on the right.

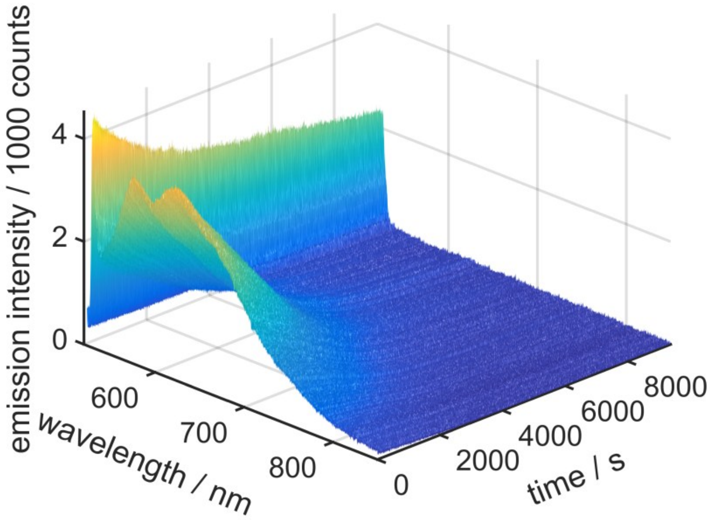

Figure S15 Raw data of the constant radius experiment for the graphene oxide sample.

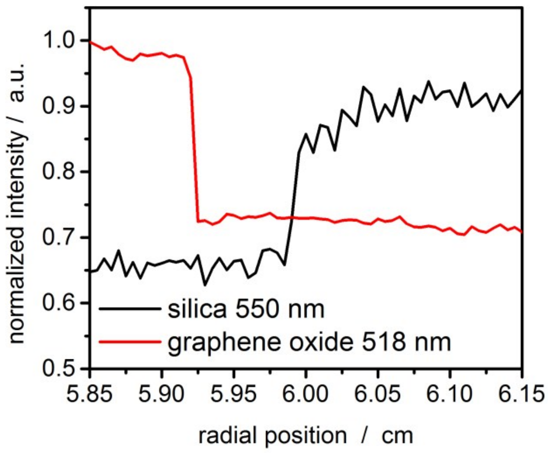

Figure S16 Normalized radial intensity scan at the end of the experiment to determine the sample meniscus. Please note that emission of the remaining free fluorophore could be used in the case of the silica particles, while the pump laser light reflected from the window-sample interface was used for the graphene oxide sample.

References

1. Zhao, H., Casillas, E., Shroff, H., Patterson, G.H. & Schuck, P. Tools for the quantitative analysis of sedimentation boundaries detected by fluorescence optical analytical ultracentrifugation. *PloS one* **8,** e77245 (2013).

2. Brautigam, C.A. in *Methods in enzymology* (Elsevier2015), pp. 109–133.

3. Schuck, P. Size-Distribution Analysis of Macromolecules by Sedimentation Velocity Ultracentrifugation and Lamm Equation Modeling. *Biophysical Journal* **78,** 1606–1619 (2000).
